# Supplementary figures and images for: MKP-1 modulates ubiquitination/phosphorylation of TLR signaling
Source: Life Sci Alliance. 2021 Sep 27;4(12):e202101137. doi: 10.26508/lsa.202101137 (PMC8500224; doi:10.26508/lsa.202101137)

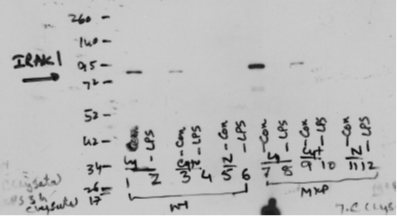

Supplement: Supplementary file 1 [file LSA-2021-01137_SdataF2.1.tif]

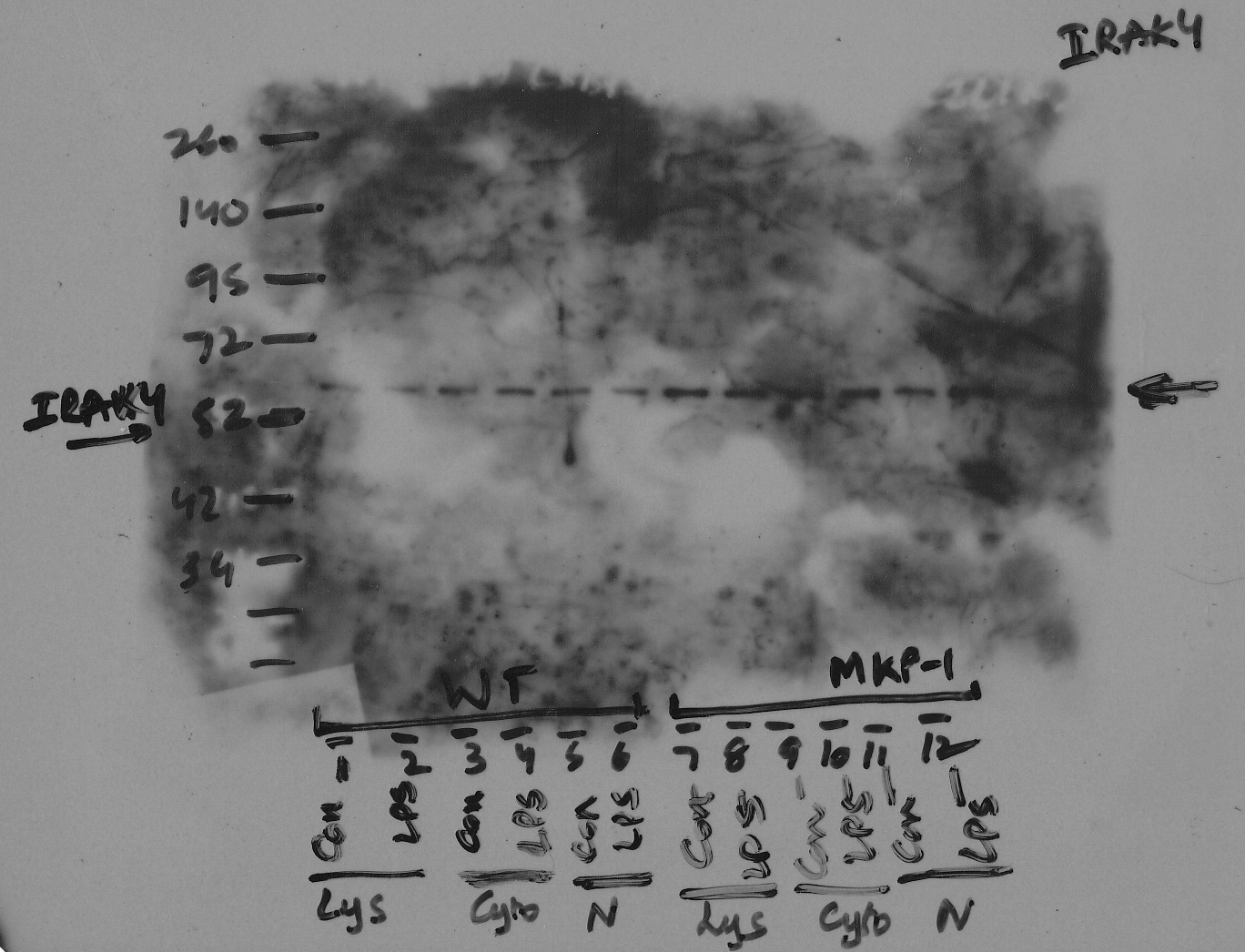

Supplement: Supplementary file 2 [file LSA-2021-01137_SdataF2.2.tif]

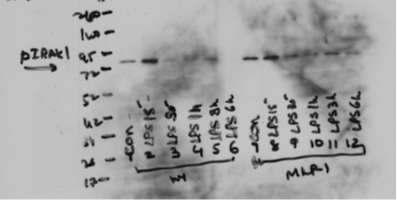

Supplement: Supplementary file 3 [file LSA-2021-01137_SdataF2.3.tif]

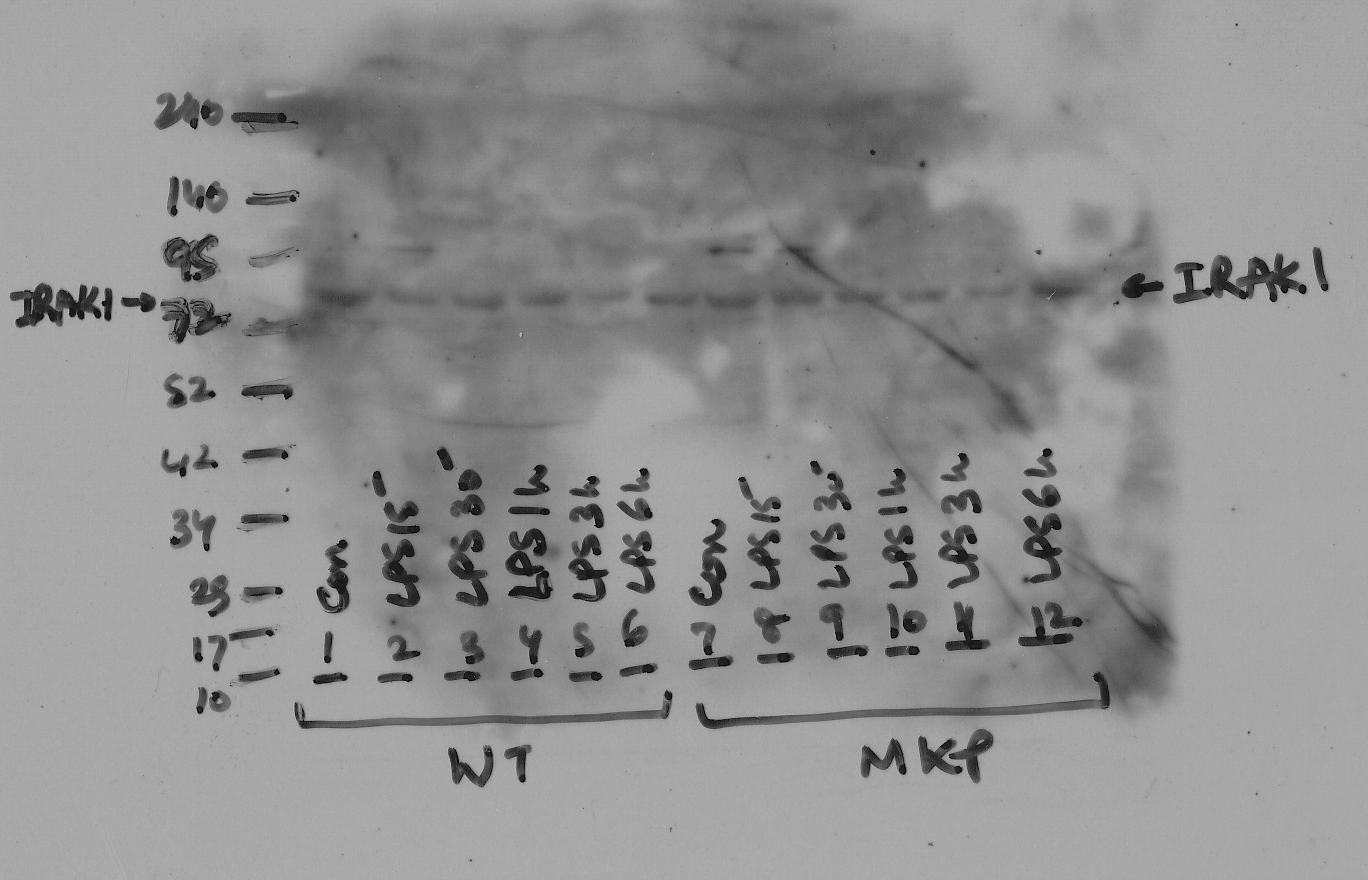

Supplement: Supplementary file 4 [file LSA-2021-01137_SdataF2.4.tif]
